# Canonical WNT Signaling Activated by WNT7B Contributes to L-HBs-Mediated Sorafenib Resistance in Hepatocellular Carcinoma by Inhibiting Mitophagy

**DOI:** 10.3390/cancers14235781

**Published:** 2022-11-24

**Authors:** Li-Juan Liu, Zhao Lv, Xing Xue, Zhong-Yuan Xing, Fan Zhu

**Affiliations:** State Key Laboratory of Virology and Hubei Province Key Laboratory of Allergy & Immunology, Department of Medical Microbiology, School of Medicine, Wuhan University, Wuhan 430071, China

**Keywords:** hepatocellular carcinoma (HCC), hepatitis B virus (HBV), large HBV surface antigen (L-HBs), WNT7B, CTNNB1, sorafenib resistance, mitophagy

## Abstract

**Simple Summary:**

Hepatitis B virus (HBV) is the main cause of hepatocellular carcinoma (HCC) morbidity and mortality. Because of chemoresistance, sorafenib, the first-line systemic therapy for advanced HCC, has minimal advantages. According to recent research, HBV plays a role in sorafenib resistance. However, the mechanisms remain unknown. WNT signaling is one of the critical signal pathways connected to cancer chemoresistance. We found that WNT7B was overexpressed in HBV-associated HCC tissues. Large hepatitis B surface antigens (L-HBs) increased canonical WNT signaling in HCC cells through WNT7B/frizzled-4 (FZD4). In HCC, WNT7B increased cell proliferation and metastasis. By decreasing mitophagy, L-HBs produced sorafenib resistance via WNT7B. The findings suggest a potential molecular target for HBV-related HCC development and chemoresistance.

**Abstract:**

Hepatocellular carcinoma (HCC) is the third leading cause of cancer death globally, with hepatitis B virus (HBV) infection accounting for over half of all cases. HBV leads to the development of HCC according to a body of literature. Our previous research and other studies also suggest that HBV causes chemotherapeutic treatment resistance, however, the mechanism is uncertain. The WNT family, which encodes secreted signaling molecules, has been linked to carcinogenesis in a variety of malignancies, including HCC. However, little is known regarding WNT7B, a WNT ligand, in the development of HCC and HBV-induced chemoresistance. In this study, the bioinformatics analysis and immunohistochemistry (IHC) staining of clinical samples revealed that WNT7B was overexpressed in HBV-associated HCC tissues versus nontumor liver tissues, which was related to HCC patient survival. Further study in vitro showed that WNT7B and its receptor frizzled-4 (FZD4) were upregulated in response to large hepatitis B surface antigens (L-HBs). L-HBs increased canonical WNT signaling in HCC cells through WNT7B/FZD4. According to functional experiments, WNT7B enhanced the cell proliferation and metastasis in HCC. In vivo and in vitro studies investigated whether L-HBs induced sorafenib resistance by WNT7B in HCC. Interestingly, L-HBs suppressed sorafenib-induced mitophagy by increasing WNT7B/CTNNB1 signaling, resulting in chemoresistance. The findings revealed that WNT7B could be a promising molecular therapeutic target as well as a predictor of sorafenib resistance in HBV-related HCC. The suppression of HBV structural proteins such as L-HBs may play a crucial role in systemic chemotherapy resistance in HBV-associated HCC.

## 1. Introduction

Due to its increased prevalence and rapid progression, hepatocellular carcinoma (HCC) is the world’s sixth most prevalent malignancy and the third most lethal cancer. HCC accounts for 8.2% of all tumor-related deaths worldwide [1]. Hepatitis B virus (HBV) is one of seven viruses recognized as Group 1 human carcinogens according to the evaluation of the International Agency for Research on Cancer (IARC) [2]. Chronic HBV infection is responsible for at least half of all HCC cases [3]. HBV has a 3.2 kb DNA genome that is largely double-stranded and contains four open reading frames that encode HBV surface antigen (HBs), core antigen (HBc), e antigen (HBe), polymerase (P), and X protein (HBx). HBs is synthesized in three forms: large (L-HBs), medium (M-HBs), and small (S-HBs), all of which surround an HBc nucleocapsid [4]. Serum HBs levels have been linked to HCC risk in patients with low viral loads [5,6].

Systemic chemotherapy is the main treatment strategy for advanced HCC. However, tolerance to chemotherapy drugs finally leads to poor prognosis in all patients [7]. Sorafenib, a multikinase inhibitor that promotes apoptosis while inhibiting tumor-cell proliferation and angiogenesis, has been licensed for the treatment of advanced liver cancer since 2007 [8]. It is an effective first-line targeted therapy for late-stage HCC, with a median overall survival of 10.7 months. However, after 18 months, the majority of patients developed sorafenib resistance [8,9]. A phase III randomized controlled study of sorafenib discovered that HBV-positive HCC patients had a worse survival benefit than HBV-negative patients [10], showing that HBV may play a vital role in a lower likelihood of survival caused by sorafenib resistance. Mechanism studies showed that HBx and HBc were correlated to chemoresistance [11,12]. However, it is unknown what role HBs play in the development of HCC and sorafenib resistance.

The WNT signaling pathway, which encompasses the CTNNB1-dependent (canonical) and CTNNB1-independent (non-canonical) pathways, is hyperactivated in HCC [13]. WNT7B, a WNT ligand, is involved in a number of cancers and contributes to drug resistance in several types of malignancies by activating canonical WNT signaling, including gemcitabine resistance in pancreatic cancer, cis-platinum resistance in cervical cancer, and doxorubicin resistance in osteosarcoma [14,15,16]. Whether WNT7B is involved in sorafenib resistance in HBV-associated HCC via canonical WNT signaling is uncertain.

Mitophagy, a type of autophagy, regulates mitochondrial homeostasis in response to cellular stresses [17]. The outer mitochondrial membrane kinase ubiquitin-dependent PTEN-induced putative kinase 1 (PINK1) is triggered by mitochondrial depolarization and recruits the cytosolic ubiquitin E3 ligase PARKIN to damaged mitochondria, resulting in mitophagy activation [18]. Sorafenib treatment has been demonstrated to generate mitochondrial stress, which induces mitophagy in order to degrade sorafenib-damaged mitochondria [19,20]. Nonetheless, the significance of mitophagy in HBV-HCC sorafenib resistance is unknown.

In this study, WNT7B was discovered to be overexpressed in HBV-associated HCC tissues and cell lines. L-HBs enhanced the expression of WNT7B and its receptor frizzled-4 (FZD4) in HCC cells. Furthermore, L-HBs activated CTNNB1/TCF-dependent proto-oncogene transcription via WNT7B/FZD4. The CCK-8 assay, foci formation assay, wound healing assay, and Transwell assay all demonstrated that WNT7B played an oncogenic role in the development of HCC. Intriguingly, L-HBs reduced sorafenib-induced apoptosis by WNT7B-inhibited mitophagy. As a result, our findings revealed that L-HBs contributed to sorafenib resistance by decreasing mitophagy mediated by the WNT7B-activated canonical WNT signal pathway. WNT7B is a potentially valuable molecular candidate for HBV-related HCC therapy. It is also a target for predicting sorafenib resistance in HBV-related HCC. HBV activity, including not only HBV DNA but also HBV structural proteins such L-HBs, plays an anti-chemotherapy effect in HCC. As a consequence, for advanced HCC patients with chronic HBV infection, a comprehensive reduction in HBV replication, including the formation of HBV structural proteins, may be beneficial.

## 2. Materials and Methods

### 2.1. Bioinformatics Analysis

The Gene Expression Omnibus (GEO) dataset GSE87630 [21], which contains 64 cases of HCC specimens with HBV infection and 30 cases of nontumoral surrounding tissue specimens, was searched for genes that were significantly dysregulated in HBV-related HCC. The platform for this work was GPL6947 (Illumina Human HT-12 V3.0 expression beadchip). The Limma package [22] of R software was used for all bioinformatics analyses, with the standard of *p*-value < 0.05. The R programming language was also used to compare gene expression patterns in tumor and nontumorous tissues. Gene Ontology (GO) annotation was performed on R software target genes to demonstrate the activities of target genes in cellular components and biological processes. Pathway analysis using the Kyoto Encyclopedia of Genes and Genomes (KEGG) was performed to uncover notable pathways connected with target genes.

The survival analysis was carried out using an online Kaplan–Meier plotter [23]. STRING bioinformatics tool was used to predict interacting proteins [24].

### 2.2. Clinical Tissues

A total of 169 HCC samples with chronic HBV infection and 17 normal liver tissues were collected. All tissue samples were obtained from the Renmin Hospital of Wuhan University (Wuhan, China). The sample collection was carried out in accordance with consensus agreements and was authorized by the ethics committee of Wuhan University, School of Medicine (Ethics No. 14011). The study adhered to the International Ethical Guidelines for Biomedical Research Involving Human Subjects (CIOMS).

### 2.3. Immunohistochemistry (IHC)

The clinical samples were fixed with 4% paraformaldehyde. The samples were then embedded in paraffin and sectioned. WNT7B was stained with the primary rabbit polyclonal antibody (PA5-103480, Thermo Fisher Scientific, Sunnyvale, CA, USA), followed by HRP conjugated goat anti-rabbit IgG (31460, Thermo Fisher Scientific). Gene expression was graded on a scale of 0–3 as follows: a score of 0 indicates that there is no membranous staining in any of the tumor cells; a score of 1 indicates that there is less than 10% of the tumor cells with any intensity or less than 30% of the tumor cells with weak intensity; a score of 2 indicates that there is staining in 10–30% of the tumor cells with moderate-to-strong intensity or staining in 30–50% of the tumor cells with weak-to-moderate intensity; a score of 3 indicates that there is staining in more than 50%. Positive samples had a score of 2 or above.

### 2.4. Plasmids Construction

Full-length cDNAs of HBV structural proteins (L-HBs, S-HBs, HBc, HBe, HBp) and human WNT7B were PCR-amplified from HepG2.2.15 cells and cloned into the pcDNA3.1(-) vector as directed by the manufacturer (V795-20, Invitrogen, Burlington, ON, USA).

WNT7B and FZD4 were knocked down using particular short hairpin RNAs (shRNA). WNT7B was targeted by the sequences 5′-CCCGAUGCCA UCAUUGUGAUU-3′ (WNT7B#1) and 5′-CAACAAGAUUCCUGGCCUA-3′ (WNT7B#2). The target sequence for FZD4 was 5′-GGUGAUGAAGAGGU GCCCUU-3′. The shRNA-coding oligonucleotides were annealed and ligated into the pSilencer 2.1-U6 neo following the manufacturer’s instructions (AM5764, Ambion, Austin, TX, USA).

### 2.5. Cell Culture and Transient Transfection 

The American Type Culture Collection (ATCC, Manassas, VA, USA) provided the HepG2, HepG2.2.15, Huh7, and HCCLM3 cells used in this work. HL-7702 was gifted by Professor Wenjie Huang at Huazhong University of Science and Technology. Cells were cultured in Dulbecco’s modified Eagle medium (2347432, Gibco BRL, Carlsbad, CA, USA) supplemented with 10% fetal bovine serum, 100 U/mL penicillin, and 100 μg/mL streptomycin sulfate at 37 °C with 5% CO_2_. When cells reached 70–90% confluence, Lipofectamine 2000 was used for transient transfection (11668019, Invitrogen).

### 2.6. RNA Extraction and Real-Time Quantitative Reverse Transcriptase-PCR (qPCR)

Total RNA was isolated from cells using TRIzol Reagent (15596026, Invitrogen). After removing genomic DNA contaminations from total RNA using DNase I (EN0521, Thermo Fisher Scientific), 1 μg of RNA was converted into cDNA using ReverTra Ace (FSK-101, TOYOBO, Osaka, Japan) according to the manufacturer’s protocol. 

qPCR was carried out in a final volume of 20 µL on the iCycler system (C1000, Bio-Rad, Hercules, CA, USA) using the SYBR Green master mixture (04913914001, Roche, Basle, Switzerland). To standardize the gene expression data, β-actin was employed as an endogenous control. Supplementary Table S1 lists the primer sequences. The reactions were incubated at 95 °C for 10 min, followed by 40 cycles of 95 °C for 10 s, 58 °C for 10 s, 72 °C for 10 s, and 60 °C for 1 min. The 2(^−ΔΔ^Ct) method for the relative quantification of gene expression was used to determine the gene expression levels.

### 2.7. Protein and Mitochondria Isolation 

After cleaning the cells with 1× PBS buffer, they were lysed using M-PER mammalian protein extraction reagent (78501, Thermo Fisher Scientific) supplemented with a Cocktail (11873580001, Roche).

Mitochondria were isolated using a commercial mitochondrial extraction kit, as directed by the manufacturer (SM0020, Solarbio, Beijing, China). 5 × 10^7^ cultivated cells were homogenized for mitochondria isolation. For mitochondria isolation, 100 μg fresh minced tissues from subcutaneous xenografts in nude mice were homogenized.

Protein concentrations were determined using the BCA protein assay kit (23250, Thermo Fisher Scientific) as directed by the manufacturer.

### 2.8. Western Blotting Analysis 

Protein extracts (30 μg) were separated in 4–12% SDS-polyacrylamide gels (80 V, 2.5 h). The proteins were electrophoretically transferred from gels to nitrocellulose membranes (10600002, GE Healthcare Life Sciences, Amersham, UK) using the TRANS-BLOT SD SEMI DRY TRANSFER CELL (BIO-RAD, 22v, 24 min). The membranes were treated with the appropriate primary antibodies and fluorescent-conjugated secondary antibodies after blocking in 5% skim milk for 60 min. The specific primary antibodies were as follows WNT7B (A7746, 1:1000, ABclonal, Wuhan, China), CTNNB1 (A19657, 1:1000, ABclonal), FZD4 (A8161, 1:1000, ABclonal), c-MYC (A1309, 1:1000, ABclonal), CCND1 (A19038, 1:1000, ABclonal), PINK1 (A7131, 1:1000, ABclonal), PARKIN (A11172, 1:1000, ABclonal), LC3B (A19665, 1:1000, ABclonal), and VDAC1 (A15735, 1:1000, ABclonal). To normalize the expression levels of different proteins, an anti-β-actin-peroxidase monoclonal antibody (A3854, Sigma-Aldrich, St. Louis, MO, USA) was utilized as an internal control. ECL reagents (34080, Millipore, Burlington, MA, USA) were used to view the bands in a Tanon 5200 MultiImage System (Tanon Science & Technology, Shanghai, China). Band intensities were quantified using ImageJ software (National Institutes of Health, Bethesda, MD, USA).

### 2.9. TOPFlash Reporter Assay

The relevant plasmids were cotransfected with either the WNT signaling reporter TOPFlash (TCF reporter plasmid) or the negative control FOPFlash (mutant, inactive TCF binding site) (17-285, Millipore) according to the manufacturer’s procedure to assess the transcriptional activity of the canonical WNT pathway.

The indicated plasmids were transiently co-transfected in Huh7 or HCCLM3 cells with either 2 µg TOPFlash or FOPFlash, and 0.5 µg pSV40-Renilla plasmid (E6911, Promega, Madison, WI, USA) as an internal control for 48 h. The firefly and Renilla luciferase activity ratio was determined using the Dual-Luciferase reporter assay system (E1960, Promega).

### 2.10. Cell Counting Kit 8 (CCK-8) Assay

Cells were plated at a density of 5 × 10^3^ cells per well in 96-well plates. Cell viability was assessed using the CCK-8 assay (Dojindo Laboratories, Kumamoto, Japan) at the given time after transient transfection and (or) drug treatment. In brief, 10 μL CCK-8 reagents were supplied to the cells and incubated for 40 min–1.5 h at 37 °C. A Multiskan FC plate reader was used to measure absorbance at 450 nm (Thermo Scientific, USA).

### 2.11. Colony Formation Assay

Soft agar dishes were made with a 0.6% agar under-layer in DMEM supplemented by 10% FCS. In the same mixture containing 0.35% agar, 2 × 10^3^ cells were plated in the up-layer. After 2–3 weeks of incubation at 37 °C with 5% CO_2_, the colonies were counted using a microscope (Olympus CH-40; Olympus, Tokyo, Japan).

### 2.12. Wound Healing Assay

In 6-well plates, cells were grown until 80% confluent. Cells were subjected to serum deprivation (DEME supplemented with 1% FBS) for a further 24 h following transfection with the relevant plasmids. The monolayer was then scratched with a 200 μL pipette tip to create the linear wounds. An Olympus CH-40 microscope was used to examine cell migration into the wound area 24 h after the wound was created. For each plate, four distinct equidistant spots were measured and averaged. The migration rate was computed for each plate as the ratio of the mean distance between both borderlines after closure to the mean distance observed at 0 h.

### 2.13. Transwell Assay

Cell invasion was measured using a 24-well Transwell with a pore membrane diameter of 8 μm (Costar, Rochester, NY, USA). Matrigel (BD Biosciences, San Diego, CA, USA) diluted to 200 μg/mL was applied to the chambers and incubated for 24 h at 37 °C. Transwell chambers were seeded with 2−6 × 10^4^ cells in the serum-free medium. The bottom chamber contained 20% FBS medium. After 48 h of incubation, the cells that had migrated through the membrane were fixed, stained with crystal violet, and counted under an Olympus CH-40 microscope. Four fields in each membrane’s four quadrants were counted and averaged.

### 2.14. Sorafenib Treatment 

Sorafenib (IS0220, Solarbio) was diluted in dimethyl sulfoxide (DMSO) to a concentration of 20 mmol/L. Sorafenib was added to the culture media in serial dilutions or at the stated concentrations either directly or after plasmid transfection. The cells were cultured for 1–3 days before being tested for IC50 using the CCK-8 assay. The cells were cultured for 24 h before being tested for additional detection.

### 2.15. Flow Cytometry 

After the floating and detached cells were collected and combined, cells were treated by an Annexin V-FITC/propidium iodide apoptosis detection kit (ZP327-1, Beijing Zoman Biotechnology, Beijing, China) according to the manufacturer’s protocol. A total of 10,000 cells per sample were analyzed via Epics Altra II cytometer (Beckman Coulter, Miami, FL, USA). A Beckman Coulter Epics Altra with Expo32 software (Beckman Coulter) was used to measure cell apoptosis.

### 2.16. Subcutaneous Xenograft Experiment

The subcutaneous xenograft experiment was utilized to assess L-HBs and WNT7B’s anti-sorafenib role in vivo. Cells were transfected with the indicated plasmids. The indicated plasmids were transfected into the cells. Sorafenib was added to the culture medium at a final concentration of 20 μmol/L after 24 h of transfection. After another 24 h, 2 × 10^7^ cells were collected and resuspended in 200 μL 0.9% sodium chloride solution before being subcutaneously injected into the flank of each 4–5-week old BALB/c-nu (nude) mouse. At regular intervals (3 days), tumor formation in mice was seen. Anesthesia was used to sacrifice mice, and tumors were dissected over a 4–6 week period. The National Institutes of Health’s Guide for the Care and Use of Laboratory Animals was followed for all animal care and handling procedures. The Animal Ethics Committee of Wuhan University, as well as the Wuhan University Center for Animal Experiment/A3 Laboratory, approved the animal experiments (Animal Using Protocol number: WP202220233).

### 2.17. Confocal Imaging 

Mitophagy was visually assessed using the colocalization of mitochondria and lysosomes [25]. To observe mitochondria, HCCLM3 cells were dyed with 200 nmol/L MitoTracker Red CMXRos (M9940, Solarbio) for 30 min at 37 °C. The cells were then stained for 60 min with 100 nmol/L LysoTracker Green DND-26 (L7400, Solarbio) to detect lysosomes. After experimenting with new cultural mediums. Images were captured using an Olympus FV3000 confocal microscope (Olympus). Fluorescences were stimulated at 578 nm (Red) and 488 nm (Green) (Green). Images were analyzed using Fluoview FV31S-SW software (Olympus) to determine MitoTracker and LysoTracker colocalization.

### 2.18. Statistical Analysis

The experiments were based on at least three separate trials. To compare qualitative variables, the Chi-squared or Fisher exact tests were used. The mean ± standard deviation (SD) or standard error of the mean (SEM) of at least three independent experiments was used to express numerical data. An unpaired Student’s *t*-test, one-way ANOVA followed by Fisher’s post hoc comparison test, or two-way ANOVA with multiple post hoc comparisons were used to compare significant differences between treatments. GraphPad Prism software was used to create the graphical representations (GraphPad, San Diego, CA, USA). *p* < 0.05 (*), *p* < 0.005 (**), *p* < 0.001 (***), and *p* < 0.0001 (****) were used to represent statistical significance.

## 3. Results

### 3.1. WNT7B Is Highly Expressed in HBV-Associated HCC and Predicts Poor Prognosis

**Figure 1:**
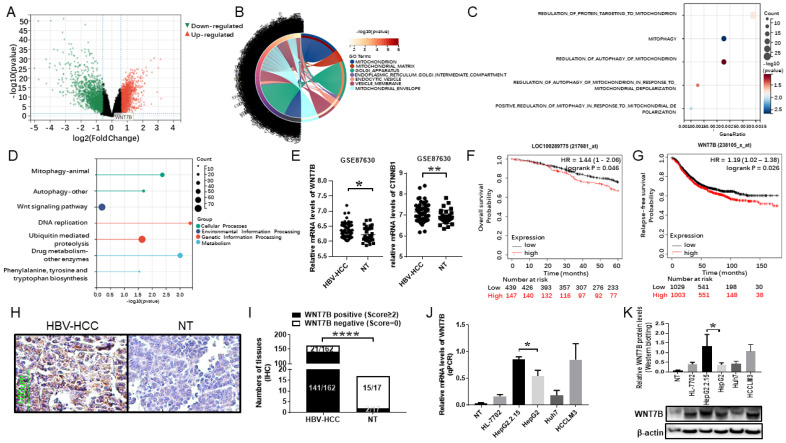
WNT7B is abundantly expressed in HBV-associated HCC and predicts a poor prognosis according to bioinformatics and clinical tissues. (**A**) The volcano plot of each gene expression profile datum was created using R software, and 9863 differentially expressed genes were discovered. (**B**,**C**) Gene Ontology (GO) analysis was performed on the differentially expressed genes based on subcellular components (**B**) and biological processes (**C**). (**D**) KEGG categorization of the differentially expressed genes in GSE87630. (**E**) The WNT7B and CTNNB1 levels in GSE87630 were determined. * *p* < 0.05, ** *p* < 0.005 (**F**,**G**) The Kaplan–Meier survival analysis of WNT7B expression and overall survival (OS) in grade 3 HCC patients (**F**) and relapse-free survival (RFS) in HCC patients (**G**) using the Kaplan–Meier Plotter. (**H**,**I**) WNT7B staining in HBV-associated HCC and nontumorous liver tissues (NTs) using immunohistochemistry. The photographs are representative (scale bar, 100 μm). The graph depicts the ratio of positive to negative WNT7B numbers in HCC and NTs. **** *p* < 0.0001 (**J**,**K**) WNT7B expression was measured in four HCC cell lines using qPCR (**J**) and Western blotting (**K**). The graphs show the mean ± SD of at least three independent experiments. * *p* < 0.05.

Detailed analyses were performed on dataset GSE87630, which included 64 cases of HCC specimens infected with HBV and 30 cases of nontumoral surrounding tissue specimens. Hierarchical clustering revealed 9863 differentially expressed genes (Figure 1A). The differentially expressed genes were mainly enriched in cellular components such as mitochondrion, Golgi apparatus, and the vesicle membrane (Figure 1B), which have distinct biological activities such as protein targeting to mitochondrion regulation and mitophagy (Figure 1C). The differentially elevated genes associated with HCC, according to KEGG enrichment analysis, were implicated in mitophagy, the WNT signaling pathway, and so on (Figure 1D). Data from the GSE87630 also revealed that WNT7B (*p* < 0.05) and CTNNB1 (*p* < 0.005) levels in HBV-associated HCC tissues were considerably greater than in nontumor liver tissues (Figure 1E), implying that WNT7B may play a significant role in the progression of HBV-associated HCC. The Kaplan–Meier survival analysis, performed using the Kaplan–Meier Plotter, an online resource for correlating gene expression with survival, showed that high WNT7B expression was significantly correlated with overall survival (OS) in grade 3 HCC patients (*p* < 0.05) and poor relapse-free survival (RFS) in HCC patients (*p* < 0.05; Figure 1F,G), suggesting that WNT7B expression may be linked to HCC recurrence.

A total of 162 HCC samples with chronic HBV infection and 17 nontumor liver tissues were gathered to validate the results of the bioinformatics analysis. WNT7B staining was positive in 141 of 162 (87.0%) HBV-HCC samples and 2 of 17 (11.8%) of NTs (*p* < 0.0001; Figure 1H,I). These findings indicate that WNT7B was considerably elevated in HBV-associated HCC.

WNT7B expression was also measured in HCC cell lines using real-time PCR (Figure 1J) and Western blotting (Figure 1K and Supplementary Figure S1). WNT7B was discovered to be expressed in all HCC cell lines. Its expression was much higher in the HCCLM3 cell line, indicating that it has spontaneous metastatic potential. Surprisingly, the WNT7B expression was higher in HepG2.2.15 than in HepG2 (*p* < 0.05). In light of the fact that the HepG2.2.15 cell line was produced from HepG2 by HBV DNA integration, resulting in sustained HBV expression and replication [26], we hypothesize that WNT7B may play a role in HBV-induced HCC.

### 3.2. L-HBs Triggers the Expression of WNT7B and Its Receptor FZD4

**Figure 2:**
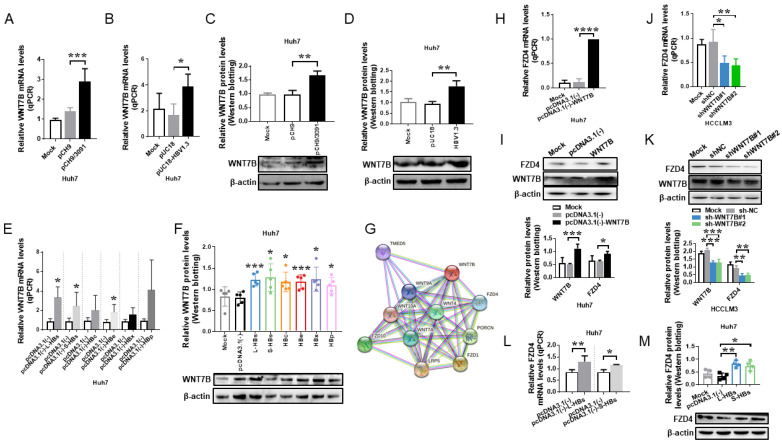
WNT7B and its receptor FZD4 are activated by large hepatitis B surface antigens (L-HBs). (**A**,**B**) qPCR analysis of WNT7B mRNA levels following pCH9/3091 or pUC18-HBV1.3 transfection. (**C**,**D**) The WNT7B protein level was investigated using a Western blotting test in response to pCH9/3091 or pUC18-HBV1.3 transfection. (**E**,**F**) WNT7B expression was measured using qPCR (**E**) and Western blotting (**F**) after the transient transfection of six HBV structural proteins (L-HBs, S-HBs, HBc, HBe, HBx, HBp). (**G**) The STRING bioinformatics tool was used to predict WNT7B interacting proteins. (**H**,**I**) FZD4 expression was measured by qPCR (**H**) and Western blotting (**I**) following WNT7B overexpression. (**J**,**K**) FZD4 expression was determined by qPCR (**J**) and Western blotting (K) following WNT7B knockdown. (**L**,**M**) The FZD4 expression level was determined by qPCR (**L**) and Western blotting (**M**) after L-HBs and S-HBs overexpression. All graphs depict the mean ± SD of at least three independent experiments. * *p* < 0.05, ** *p* < 0.005, *** *p* < 0.001, **** *p* < 0.0001.

To test whether HBV regulates WNT7B expression in HCC cells, we transfected Huh7 cells with the HBV replication plasmids, pCH9/3091 and PUC18-HBV1.3 plasmids, which contain 1.1 and 1.3 copies of the HBV genome, respectively. Real-time PCR (Figure 2A,B) and Western blotting (Figure 2C,D, and Supplementary Figure S1) revealed that WNT7B levels were higher in pCH9/3091 and HBV1.3 transfected cells than in control cells, implying that HBV infection increases WNT7B expression in HCC cells.

Six plasmids encoding HBV structural proteins (L-HBs, S-HBs, HBc, HBe, HBx, HBp) were produced and transiently transfected into Huh7 cells to further identify the HBV structural proteins that regulate WNT7B expression. WNT7B expression was determined using real-time PCR and Western blotting. WNT7B mRNA (*p* < 0.05) and protein levels (*p* < 0.001) were considerably enhanced in L-HBs-transfected Huh7 cells compared to the negative control, as illustrated in Figure 2E,F, and Supplementary Figure S1. These results suggest that L-HBs may increase WNT7B expression in HCC.

FZD genes encode seven transmembrane-type WNT receptors that transduce WNT signals. All known and predicted WNT7B interacting proteins were predicted using the STRING database. FZD4 was identified as one of the receptors that may interact with WNT7B, with a predicted functional partners score of 0.912 (Figure 2G), which was proven in endothelial cells [27]. The FZD4 receptor was shown to be upregulated at both the mRNA and protein levels after WNT7B overexpression (Figure 2H,I). In reverse, the knockdown of WNT7B reduced the expression of FZD4 (Figure 2J,K). Furthermore, both L-HBs and S-HBs can increase the mRNA and protein levels of FZD4 in Huh7 cells. However, L-HBs showed a more significant impact on FZD4 (Figure 2L,M, and Supplementary Figure S1). These findings imply that L-HBs promote WNT7B and its receptor FZD4 expression in HCC cells.

### 3.3. L-HBs-Activated CTNNB1/TCF-Mediated Transcriptional Activity via WNT7B/FZD4 in HCC Cells

**Figure 3:**
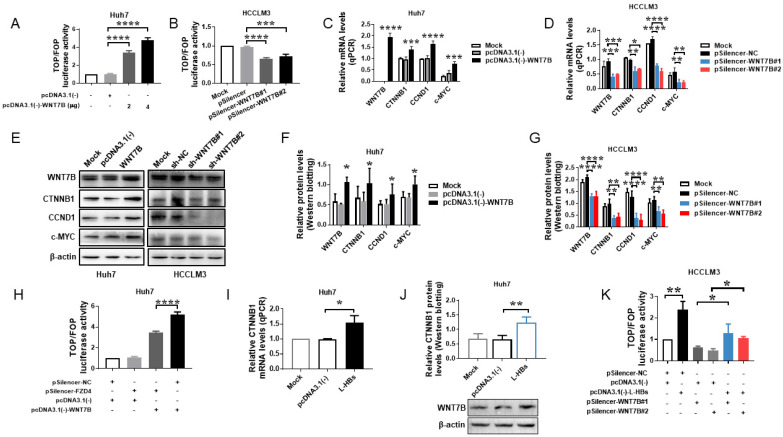
Large hepatitis B surface antigens (L-HBs) stimulate CTNNB1/TCF-mediated transcriptional activity in HCC cells via WNT7B/FZD4. (**A**,**B**) The TOP/FOP luciferase reporter assay was used to assess the effect of WNT7B on CTNNB1/TCF-mediated transcription activity. (**C**–**G**) CTNNB1, CCND1, and c-MYC mRNA and protein levels were measured using qPCR (**C**,**D**) or Western blotting (**E**–**G**) following WNT7B overexpression or knockdown. (**H**) Detection of FZD4 on WNT7B-mediated canonical WNT signaling activity by TOP/FOP luciferase reporter assay. (**I**,**J**) Regulation of CTNNB1 expression by L-HBs was determined using qPCR (I) and Western blotting (**J**). (**K**) The TOP/FOP luciferase reporter assay was used to assess the involvement of L-HBs in CTNNB1/TCF-mediated transcription activity. All graphs show the mean ± SD of at least three independent experiments. * *p* < 0.05, ** *p* < 0.005, *** *p* < 0.001, **** *p* < 0.0001.

WNT7B has been shown to activate the canonical WNT signal pathway in a variety of malignancies [14,28,29]. Bioinformatics analysis using GSE87630 showed that CTNNB1 levels were increased in HBV-associated HCC tissues (Figure 1E), suggesting that canonical WNT signaling may be activated in HBV-associated HCC. The TOP/FOP luciferase reporter assay was used to evaluate the changes in CTNNB1/TCF-mediated transcription to determine whether WNT7B activated the canonical WNT signal pathway in HCC cells. WNT7B overexpression significantly increased TOP/FOP luciferase activity by 3.4-fold (2 μg) and 4.7-fold (4 μg) compared to the empty vector control (*p* < 0.0001; Figure 3A). WNT7B knockdown decreased TOP/FOP luciferase activity in HCCLM3 cells by 32.1% (pSilencer-WNT7B#1; *p* < 0.0001) and 25.9% (pSilencer-WNT7B#2; *p* < 0.001) compared to the pSilencer-NC control (Figure 3B), indicating that WNT7B can up-regulate CTNNB1/TCF-dependent transcriptional activation in HCC cells. Furthermore, CTNNB1 expression levels, as well as those of two CTNNB1 downstream targets, CCND1 and c-MYC, were also determined. WNT7B overexpression increased CTNNB1, CCND1, and c-MYC mRNA and protein levels in Huh7 cells (Figure 3C,E,F). WNT7B knockdown significantly reduced the mRNA and protein levels of CTNNB1, CCND1, and c-MYC in HCCLM3 cells (Figure 3D,E,G, and Supplementary Figure S1). WNT7B can activate the classical WNT pathway in HCC cells, according to these findings.

The function of FZD4 in WNT7B-induced CTNNB1/TCF-mediated transcriptional activity was also determined using the TOP/FOPFlash system. The outcomes demonstrated that WNT7B elevated the TOP/FOP luciferase activity, which was significantly reduced by FZD4 knockdown by 64.7% (*p* < 0.0001; Figure 3H). These data suggested that WNT7B-induced canonical WNT signaling activity in Huh7 cells was mediated by FZD4.

Overexpression of L-HBs increased the mRNA and protein levels of CTNNB1 in Huh7 cells, as shown in Figure 3I,J, implying that L-HBs may activate the WNT7B/CTNNB1 pathway. Using the TOP/FOPFlash assay, we discovered that L-HBs significantly increased TOP/FOP luciferase activity (*p* < 0.001), indicating that L-HBs can activate canonical WNT signaling in HCC cells. Furthermore, L-HBs enhanced TOP/FOP luciferase activity, which was decreased by WNT7B knockdown (*p* < 0.05; Figure 3K). These results suggest that L-HBs activate CTNNB1/TCF-mediated transcriptional activity via WNT7B.

### 3.4. WNT7B Induces the Malignization of HCC

**Figure 4:**
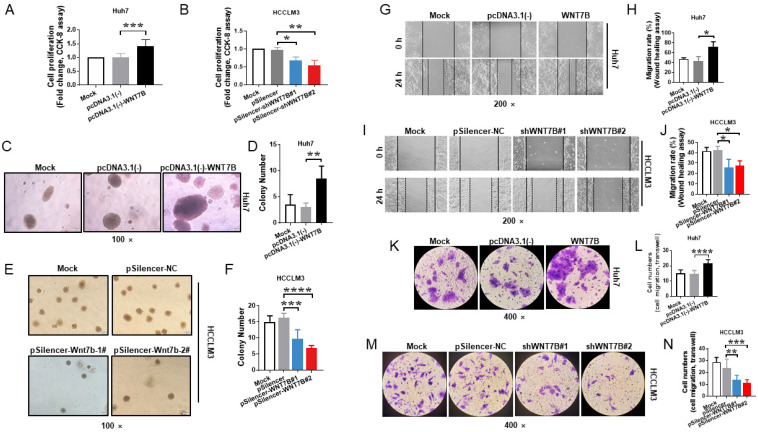
WNT7B causes the malignant properties of HCC. (**A**,**B**) The CCK-8 assay was used to determine WNT7B’s effect on cell proliferation. (**C**–**F**) The colony formation assay was utilized to investigate WNT7B’s effect on cell transformation. Representative images are shown (magnification, 100×) (**G**–**J**) The wound healing test was used to study the role of WNT7B in cell migration. Representative images are shown (magnification, 200×). (**K**–**N**) The role of WNT7B on cell invasion was studied using the Transwell assay. Representative images are shown (magnification, 400×). All graphs reflect the mean ± SD of at least three independent experiments. * *p* < 0.05, ** *p* < 0.005, *** *p* < 0.001, **** *p* < 0.0001.

The CCK-8 assay in Figure 4A revealed that WNT7B overexpression greatly increased the proliferation rate of Huh7 cells (*p* < 0.001). WNT7B knockdown in HCCLM3 cells dramatically lowered the proliferation rate relative to the controls (*p* < 0.05; Figure 4B), demonstrating that WNT7B enhanced cell proliferation. 

The Huh7-WNT7B cells generated colonies 2.83-fold more than the control (*p* < 0.005; Figure 4C,D). This indicated that WNT7B has oncogenic potential in HCC cells. The fact that it formed fewer foci following WNT7B knockdown in HCCLM3 cells proved this from another perspective (*p* < 0.001; Figure 4E,F).

When compared to the controls, WNT7B promoted wound healing in Huh7 cells (*p* < 0.05; Figure 4G,H). WNT7B knockdown, on the other hand, strongly inhibited wound closure in HCCLM3 cells (*p* < 0.05; Figure 4I,J). The invasive potential of WNT7B was confirmed using Transwell chambers with Matrigel. Cell invasion was found to be enhanced in Huh7-WNT7B cells (*p* < 0.0001; Figure 4K,L). WNT7B knockdown, in contrast, significantly reduced cell invasion in HCCLM3 cells (*p* < 0.005; Figure 4M,N).

The findings suggested that WNT7B could improve HCC cell proliferation, migration, and invasion.

### 3.5. L-HBs Participates in Sorafenib Resistance via WNT7B

**Figure 5:**
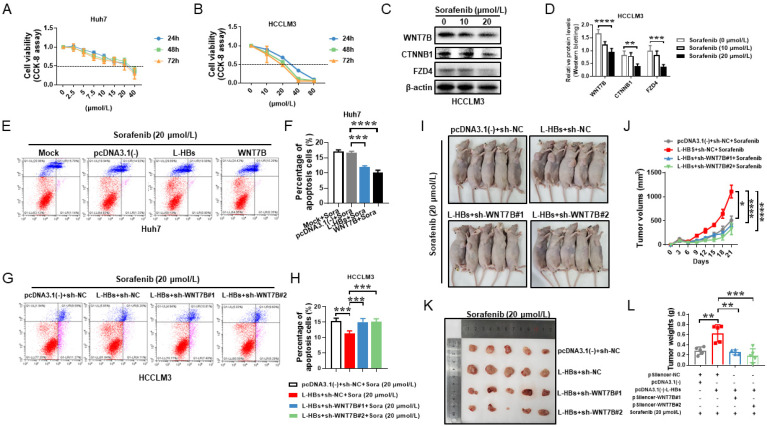
Sorafenib resistance is mediated by large hepatitis B surface antigens (L-HBs) via WNT7B. (**A**,**B**) In Huh7 and HCCLM3, cell viability was evaluated using the CCK-8 assay after treatment with a variety of sorafenib doses. (**C**,**D**) WNT7B, CTNNB1, and FZD4 expression were detected in HCCLM3 cells after sorafenib treatment by Western blotting. The levels of expression were quantified and given as mean ± SD of at least three independent experiments. (**E**–**H**) Flow cytometry was used to assess cell apoptosis rates using Annexin V-FITC/propidium iodide. A total of 10,000 cells were counted per sample for apoptosis analysis. The ratios of apoptotic cells are represented as the mean ± SD of four independent experiments. (**I**,**K**) The graph depicts the development of xenograft tumors in nude mice. (**J**,**L**) The graph shows the mean ± SEM of tumor volume (**I**) and the mean ± SD of tumor weight (**L**) induced by indicated plasmids-transfected HCCLM3 cells of five independent experiments. * *p* < 0.05, ** *p* < 0.005, *** *p* < 0.001, **** *p* < 0.0001.

L-HBs strongly activated WNT7B/CTNNB1 in HCC cells, according to our findings. WNT7B is involved in chemoresistance in several types of cancers [14,15,16], but there is no evidence that WNT7B causes sorafenib resistance in HCC. HBs levels in serum have been linked to HCC risk in patients with low viral loads [5,6]. It is currently uncertain what role L-HBs play in sorafenib resistance in HCC. Sorafenib’s cytotoxicity on Huh7 and HCCLM3 cell lines was first investigated. The results indicated that sorafenib significantly inhibited the viability of Huh7 and HCCLM3 cells in a concentration-dependent manner (Figure 5A,B).

WNT7B, FZD4, and CTNNB1 protein levels were considerably decreased in HCCLM3 cells treated with sorafenib in a concentration-dependent manner when compared to DMSO-treated cells, as shown in Figure 5C,D. Flow cytometry revealed that L-HBs or WNT7B reduced the cytotoxicity of sorafenib (20 μmol/L) by approximately 28.3% and 39.6%, respectively, compared to the negative control (Figure 5E,F, *p* < 0.001). The findings suggest that L-HBs and WNT7B might both protect HCC cells from sorafenib-induced cell death. 

Flow cytometry and xenograft in nude mice were used to assess whether L-HBs induced sorafenib resistance via WNT7B in HCC cells. Flow cytometry revealed that knocking down WNT7B expression restored L-HBs protection against sorafenib-induced apoptosis by approximately 1.3-fold (*p* < 0.001; Figure 5G,H). Overexpression of L-HBs in sorafenib-treated HCCLM3 cells increased xenograft sizes and weights compared to the negative control in the xenograft model. However, knocking down WNT7B in sorafenib-treated HCCLM3 cells drastically decreased tumor sizes and weights, which were boosted by L-HBs (Figure 5I–L). These findings imply that L-HBs mediate sorafenib resistance via WNT7B.

### 3.6. L-HBs Contributes to Sorafenib Resistance by Suppressing Mitophagy via WNT7B

**Figure 6:**
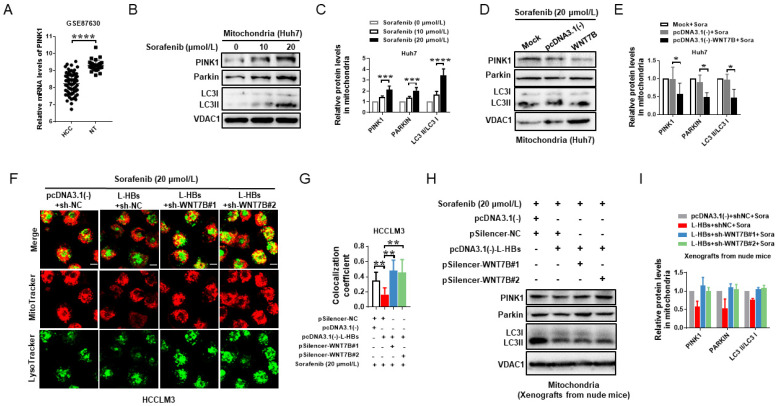
Large hepatitis B surface antigens (L-HBs) inhibit sorafenib-induced mitophagy via WNT7B in HCC. (**A**) PINK1 expression in HBV-related HCC and nontumor liver tissues were analyzed in GSE87630. (**B**,**D**,**H**) Examination of mitochondria proteins by Western blotting. (**C**,**E**,**I**) The expression of PINK1, PARKIN, LC3I, and LC3II was quantified and represented as mean ± SD. (**F**) Representative fluorescent images of MitoTracker, LysoTracker, and MitoTracker/LysoTracker colocalization using laser confocal scanning microscopy (Scale bar, 50 μm). (**G**) Quantitative analysis of MitoTracker/LysoTracker colocalization coefficient. * *p* < 0.05, ** *p* < 0.005, *** *p* < 0.001, **** *p* < 0.0001.

The KEGG enrichment analysis of differentially expressed genes in GSE87630 found that the genes associated with HCC were involved in mitophagy. The most extensively researched mitophagy route was PINK1/PARKIN. According to the GSE87630 data, the expression of PINK1 in HBV-related HCC was significantly lower than in nontumor liver tissues (*p* < 0.0001; Figure 6A), implying that mitophagy activity may be diminished in HBV-related HCC. We then investigated whether PINK/PARKIN-mediated mitophagy was induced in sorafenib-treated HCC cells. As shown in Figure 6B,C, and Supplementary Figure S2, there was a significant increase in the accumulation of mitochondrial proteins (PINK1, PARKIN, LC3-II) in the mitochondria of sorafenib-treated Huh7 cells in a concentration-dependent manner, indicating that mitophagy was hyperactivated in the Huh7 cells when compared to the control.

We then investigated the involvement of WNT7B in sorafenib-induced mitophagy in HCC cells. The mitochondrial proteins (PINK1, PARKIN, LC3-II) in WNT7B overexpressed sorafenib-treated Huh7 cells were determined. WNT7B overexpression suppressed the protein levels of PINK1, PARKIN, and LC3B-II in the mitochondria of Huh7 cells treated with sorafenib compared to the negative control (Figure 6D,E, and Supplementary Figure S2), revealing that WNT7B suppresses mitophagy activity in sorafenib-treated Huh7 cells.

**Figure 7:**
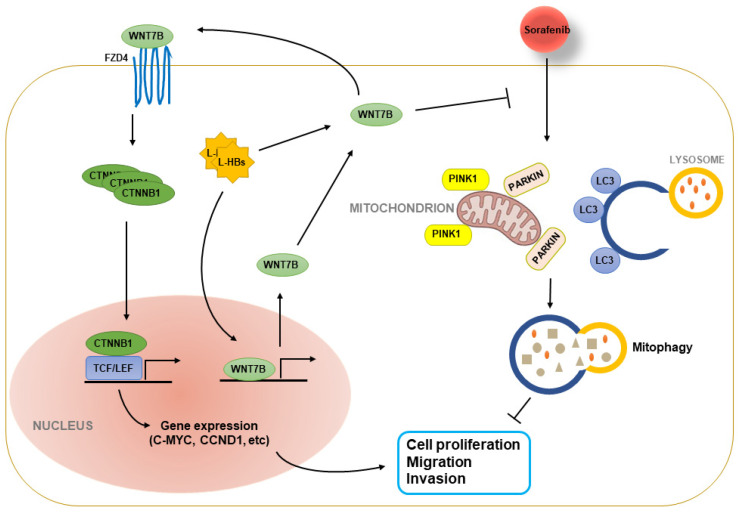
Schema depicts the process through which large hepatitis B surface antigens (L-HBs) stimulate WNT7B/CTNNB1 signaling and contribute to sorafenib resistance in HCC. L-HBs boosted canonical WNT signaling in HCC cells via WNT7B/FZD4 and the elevation of downstream proteins (such as c-MYC and CCND1) levels. In HCC, sorafenib therapy triggered PINK1/PARKIN-dependent mitophagy. L-HBs caused sorafenib resistance by reducing mitophagy via WNT7B.

To determine the impact of L-HBs on mitophagy, the cells were stained with MitoTracker Red and LysoTracker Green, which mark mitochondria and lysosomes, respectively. L-HBs overexpression dramatically reduced the colocalization of mitochondria and lysosomes in sorafenib-treated HCCLM3 cells, as demonstrated in Figure 6F,G. WNT7B knockdown, on the other hand, significantly improved the colocalization of mitochondria and lysosomes in HCC cells with L-HBs overexpression and sorafenib therapy. L-HBs also reduced mitochondrial proteins (PINK1, PARKIN, and LC3-II) in xenografts formed by sorafenib-treated HCCLM3 cells. WNT7B knockdown boosted mitochondrial protein expression in xenografts produced by HCC cells with L-HBs overexpression and sorafenib therapy (Figure 6H, and Supplementary Figure S2). The findings indicate that L-HBs play a role in sorafenib resistance by suppressing mitophagy mediated by WNT7B (Figure 7).

## 4. Discussion

HCC is a serious cancer with a slow beginning, invasive growth, a high recurrence rate, and a high mortality rate. The median survival of patients with advanced HCC is less than one year. Systemic chemotherapy is the only option for such patients [1,7]. Despite advances in chemotherapies, the median overall survival remained less than a year [30]. Chemoresistance mechanisms include dysregulated efflux and inflow transporters, metabolic reprogramming, an abnormal immunological microenvironment, extracellular vesicles, and others [31,32,33]. HBV is also linked to chemoresistance, according to recent limited data and our earlier studies [10,12,34]. Our data in this article show sorafenib resistance in response to L-HBs. We also discovered that L-HBs stimulated the WNT7B-mediated canonical WNT signaling pathway, which aided in the development of HCC and sorafenib resistance by suppressing mitophagy. 

Research on predictive and prognostic HCC biomarkers and their reaction to chemoresistance is required in order to improve patient outcomes and achieve therapeutic advances in systemic chemotherapy. Sadly, no biomarkers for either systemic treatment were reported to predict responses in HCC [30]. In this investigation, we discovered that HBV-associated HCC tissues and HBV-replicating cell lines both had high levels of WNT7B expression, indicating that WNT7B may be a critical factor in HBV-induced HCC. Patients with late-stage HCC who tested positive for WNT7B had a shorter overall survival. When combined with the finding that individuals with late-stage HCC can only get systemic treatment [7], it is possible that WNT7B is implicated in chemoresistance.

WNT signaling pathways, particularly canonical WNT signaling, have been demonstrated to be regularly active in HBV-induced HCC [35,36]. The canonical WNT signaling pathway is activated by inhibiting the phosphorylation-dependent degradation of the transcriptional coactivator CTNNB1. CTNNB1 accumulates in the cytoplasm and then translocates to the nucleus, where it interacts with TCF/LEF family members, initiating a variety of intracellular signaling cascades [13]. The WNT/CTNNB1 signaling pathway can be activated by HBx, which increases the expression and stability of WNT pathway target genes [37,38]. S-HBs has also been shown to raise the levels of LEF-1, c-MYC, and CCND1, all of which are downstream from the WNT pathway [39]. Here, we discovered that the HBV whole genome and L-HBs can increase WNT7B and FZD4 expression. L-HBs can activate canonical WNT signaling in HCC cells via WNT7B, as evidenced by increased downstream genes c-MYC and CCND1. Our results suggest that L-HBs also activated the canonical WNT pathway mediated by WNT7B. A study [40] found that the T-complex protein-1 ring complex subunit (also known as TCP1) activates the WNT7B-mediated canonical WNT pathway in HCC, validating our findings that WNT7B can activate CTNNB1-dependent WNT signaling in HCC.

WNT7B is involved in a number of cancers. Wnt7B knockdown inhibits pancreatic cancer stem cell proliferation [14]. In breast cancer cells, WNT7B stimulates cell proliferation and migration [28]. WNT7B mRNA from extracellular vesicles is transported to and modulates human umbilical vein endothelial cells toward higher proliferative and angiogenesis [29]. Our findings demonstrate that WNT7B overexpression can increase the proliferation, malignant transformation, and invasion of HCC cells, implying an oncogenic role for WNT7B in HCC. Because HBs has been linked to an HCC risk in patients with low viral loads [5,6], it is believed that L-HBs caused HCC development by hyperactivating WNT7B-mediated canonical WNT signaling.

Sorafenib is an effective first-line therapy for late-stage HCC [41]. However, due to the early onset of sorafenib resistance, most patients did not benefit long-term [42]. Clinical trial data reveal that HBV infection is related to a decreased response to sorafenib treatment [10]. HBV-replicating hepatoma cells are also less responsive to sorafenib treatment [43,44]. Mechanism investigations revealed that HBx depletion reduces the effects of sorafenib resistance in HCC [37]. In this work, we discovered that L-HBs prevented sorafenib-induced apoptosis and increased the growth of xenografts in nude mice, showing that L-HBs play a role in sorafenib resistance in HCC.

Canonical WNT signaling was also implicated in sorafenib resistance [45,46]. A fraction of HBV-replicating cells in HBV-associated HCC increases WNT signaling and is resistant to sorafenib [47]. One study found that cutting down GSK3, a critical component of the CTNNB1 destruction complex, eliminates the inhibitory effects of HBV X protein depletion on sorafenib resistance in HCC [37], showing that canonical WNT signaling is involved in sorafenib resistance in HBV-related HCC. Our findings demonstrated that L-HBs produced sorafenib resistance via WNT7B, revealing a novel mechanism whereby HBV induces sorafenib resistance by modulating the WNT signaling pathway.

Mitophagy activation has a dual role in modulating sorafenib sensitivity [42]. In Chen et al.’s study, ketoconazole, which resulted in PARKIN mitochondrial translocation and excessive mitophagy, sensitized HCC cells to sorafenib treatment [48]. Another study [49] found hypoxia-induced sorafenib-resistant HCC cells with hyperactivated mitophagy mediated by PINK1/PARKIN. In sorafenib-treated HCC cells, PINK/PARKIN-mediated mitophagy was induced, according to our findings. PINK1 was also found to be downregulated in HBV-HCC tissues. In HCC, L-HBs induced sorafenib resistance by blocking PINK1/PARKIN-mediated mitophagy via WNT7B. As a result, we postulated that WNT7B/CTNNB1 signaling activation in HBV-associated HCC contributed to the development of HCC and that L-HBs generated sorafenib resistance by reducing mitophagy.

## 5. Conclusions

According to our findings, increased WNT7B expression was associated with HBV-induced HCC and predicted a poor prognosis in HCC. L-HBs boosted WNT7B and its receptor FZD4 expression, as well as WNT7B/FZD4-mediated canonical WNT signaling activity in HCC cells. WNT7B increased HCC cell proliferation, malignancy, and metastasis. L-HBs suppressed sorafenib-induced mitophagy via increasing WNT7B/CTNNB1 signaling, resulting in sorafenib resistance. Because of WNT7B’s aggressive role in HBV-mediated hepatocarcinogenesis and L-HBs-mediated sorafenib resistance, it is appealing and effective for improving drug resistance in HBV-induced HCC. This study will also help expand the mechanism of HBs-mediated chemoresistance in HCC.

## Data Availability

The data presented in this study are available in this article.

## Supplementary Materials

The following supporting information can be downloaded at: https://www.mdpi.com/article/10.3390/cancers14235781/s1, Figure S1: Original blots in Figure 1, Figure 2 and Figure 3; Figure S2: Original blots in Figure 6; Table S1: Primer sequences used for real-time quantitative reverse transcriptase-PCR.
