# Supplementary material for: Canonical WNT Signaling Activated by WNT7B Contributes to L-HBs-Mediated Sorafenib Resistance in Hepatocellular Carcinoma by Inhibiting Mitophagy
Source: Cancers (Basel). 2022 Nov 24;14(23):5781. doi: 10.3390/cancers14235781 (PMC9741164; doi:10.3390/cancers14235781)

Supplementary Figure S1

Fig. 1K, panel #1 WNT7B

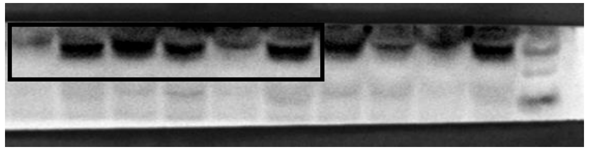

Fig. 2C, panel #1 WNT7B

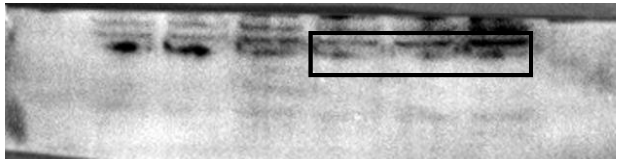

Fig. 2D, panel #1 WNT7B

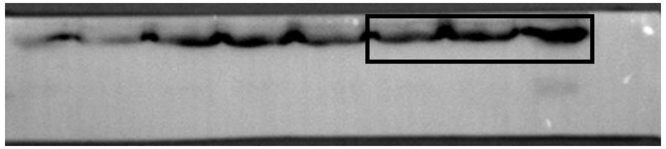

Fig. 2F, panel #1 WNT7B

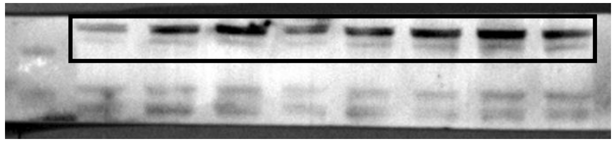

Fig. 2M, panel #1 FZD4

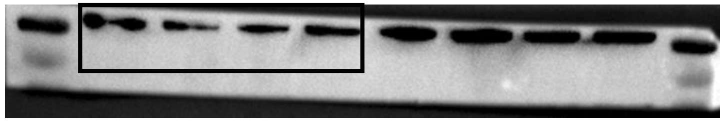

Fig. 3J, panel #1 WNT7B

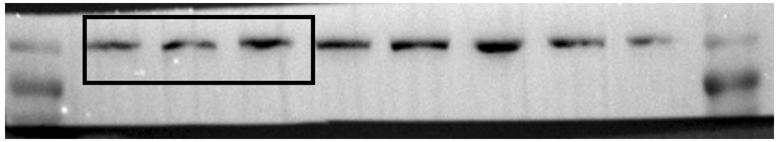

Fig. 1K, panel #2 β-actin

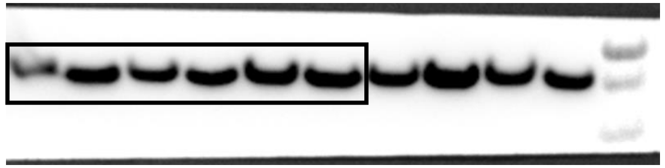

Fig. 2C, panel #2 β-actin

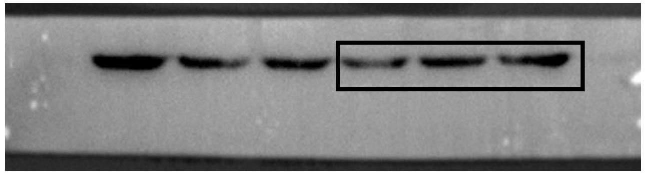

Fig. 2D, panel #2 β-actin

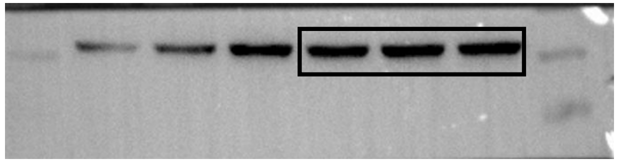

Fig. 2F, panel #2 β-actin

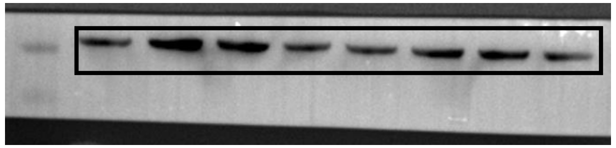

Fig. 2M, panel #2 β-actin

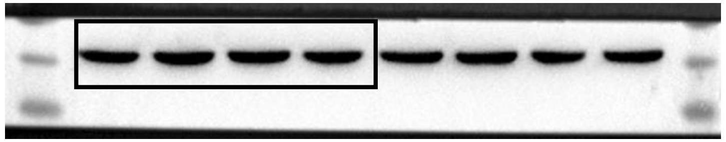

Fig. 3J, panel #2 β-actin

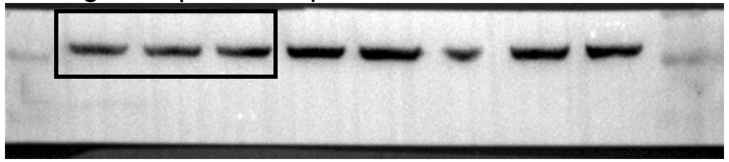

Supplementary Figure S2

Fig. 6B, panel #1 PINK1

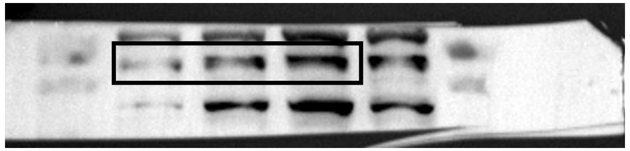

Fig. 6B, panel #2 PARKIN

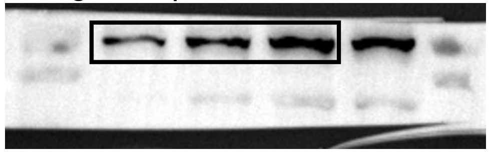

Fig. 6B, panel #3 LC3B

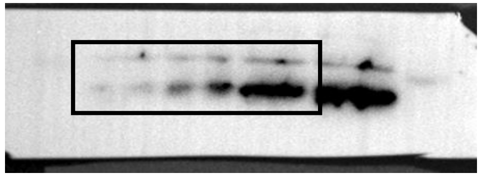

Fig. 6B, panel #4 VDAC1

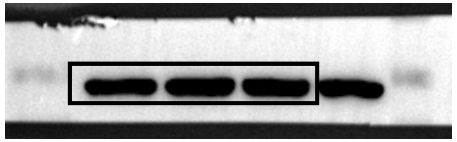

Fig. 6D, panel #1 PINK1

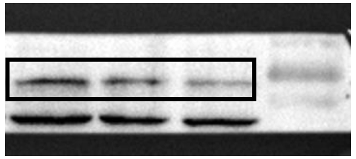

Fig. 6D, panel #2 PARKIN

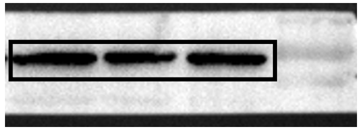

Fig. 6D, panel #3 LC3B

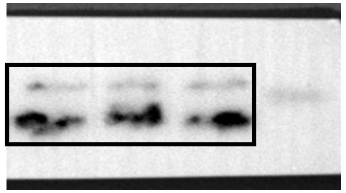

Fig. 6D, panel #4 VDAC1

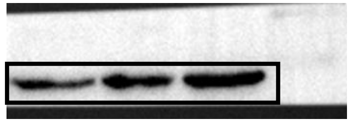

Fig. 6H, panel #1 PINK1

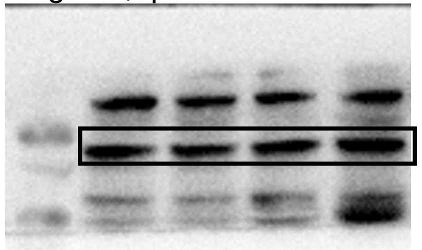

Fig. 6H, panel #2 PARKIN

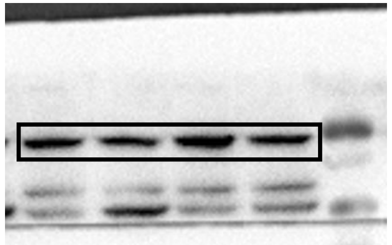

Fig. 6H, panel #3 LC3B

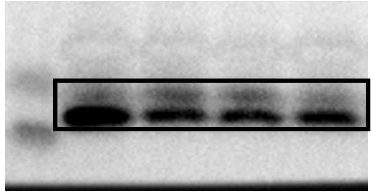

Fig. 6H, panel #4 VDAC1

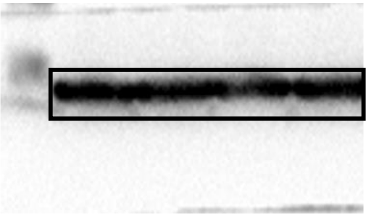

Supplement: Supplementary file 1 [file cancers-14-05781-s001.zip › cancer-2028305-supplementary figures.pdf]
